# PAI‐1 5G/5G genotype is an independent risk of intracranial hemorrhage in post‐lysis stroke patients

**DOI:** 10.1002/acn3.50923

**Published:** 2019-10-21

**Authors:** István Szegedi, Attila Nagy, Edina G. Székely, Katalin R. Czuriga‐Kovács, Ferenc Sarkady, Levente I. Lánczi, Ervin Berényi, László Csiba, Zsuzsa Bagoly

**Affiliations:** ^1^ Faculty of Medicine Department of Neurology Doctoral School of Neuroscience University of Debrecen 22 Móricz Zsigmond krt. Debrecen 4032 Hungary; ^2^ Faculty of Public Health Department of Preventive Medicine University of Debrecen 26 Kassai út Debrecen 4032 Hungary; ^3^ Faculty of Medicine Department of Laboratory Medicine Division of Clinical Laboratory Sciences University of Debrecen 98 Nagyerdei krt. Debrecen 4032 Hungary; ^4^ Faculty of Medicine Department of Radiology University of Debrecen 98 Nagyerdei krt. Debrecen 4032 Hungary; ^5^ MTA‐DE Cerebrovascular and Neurodegenerative Research Group 22 Móricz Zsigmond krt. Debrecen 4032 Hungary

## Abstract

**Objective:**

Thrombolysis by recombinant tissue plasminogen activator (rt‐PA) is the main pharmacological therapy in acute ischemic stroke (IS); however, it is only effective in a subset of patients. Here we aimed to investigate the role of plasminogen activator inhibitor‐1 (PAI‐1), an effective inhibitor of t‐PA, and its major polymorphism (PAI‐1 4G/5G) in therapy outcome.

**Methods:**

Study population included 131 consecutive IS patients who all underwent thrombolysis. Blood samples were taken on admission, 1 and 24 h after rt‐PA infusion. PAI‐1 activity and antigen levels were measured from all blood samples and the PAI‐1 4G/5G polymorphism was determined. Clinical data including NIHSS were registered on admission and day 1. ASPECTS was assessed using CT images taken before and 24 h after thrombolysis. Intracranial hemorrhage (ICH) was classified according to ECASS II. Long‐term outcome was defined 90 days post‐event by the modified Rankin Scale (mRS).

**Results:**

PAI‐1 activity levels dropped transiently after thrombolysis, while PAI‐1 antigen levels remained unchanged. PAI‐1 4G/5G polymorphism had no effect on PAI‐1 levels and did not influence stroke severity. PAI‐1 activity/antigen levels as measured on admission were significantly elevated in patients with worse 24 h ASPECTS (<7). Logistic regression analysis including age, sex, NIHSS on admission, BMI, history of arterial hypertension, and hyperlipidemia conferred a significant, independent risk for developing ICH in the presence of 5G/5G genotype (OR:4.75, 95%CI:1.18–19.06). PAI‐1 levels and PAI‐1 4G/5G polymorphism had no influence on long‐term outcomes.

**Interpretation:**

PAI‐1 5G/5G genotype is associated with a significant risk for developing ICH in post‐lysis stroke patients.

## Introduction

Ischemic stroke (IS) is a common vascular disease with high morbidity and mortality.^1^ Although mechanical thrombectomy has revolutionized stroke care in the past decade,^2^ the most commonly used pharmacological therapy of IS is still intravenous (i.v.) thrombolysis with recombinant tissue plasminogen activator (rt‐PA). With the extended time window of 4.5 h,^3^ about half of the patients receiving this therapy attain total or nearly total neurological recovery at 3 months.^4^

Although the relative benefit of i.v. t‐PA is unquestionable for selected patients with acute IS, unfortunately, in a large proportion of patients neurological improvement fails and about 6–8% develop hemorrhagic transformation after thrombolysis as a side effect.^5^ In order to improve safety and outcome of i.v. rt‐PA therapy, there is a growing interest in finding new biomarkers as potential predictors of post‐lysis intracranial bleeding and treatment failure.

PAI‐1 is a serine protease inhibitor that plays an essential role in the regulation of the fibrinolytic system. As the most important inhibitor of t‐PA, the relative balance between PAI‐1 and t‐PA plays an important regulatory role in fibrinolysis.^6^ Elevated PAI‐1 levels have been long identified as risk factor for cardiovascular disease and stroke.^6^, ^7^, ^8^ Surprisingly, much less evidence is available on changes in PAI‐1 levels during thrombolysis in stroke patients and its association with outcomes.

PAI‐1 levels are regulated by a number of factors, for example inflammation, obesity, circadian rhythm, and genetic factors.^6^, ^9^, ^10^ Several polymorphisms within the PAI‐1 gene have been described to influence PAI‐1 levels, of which the most studied is the −675 4G/5G polymorphism of the promoter region (rs1799889). It has been shown that both alleles of 4G/5G polymorphism contain a binding site for a transcription activator, while the 5G allele also contains a binding site for a transcription repressor that partially overlaps with the activator‐binding site.^11^, ^12^ Therefore, individuals with 4G/4G genotype have the highest, while those with 5G/5G genotype have the lowest circulating PAI‐1 levels.^13^ An association between the 4G/5G polymorphism and the risk of cardiovascular or cerebrovascular disease has been described in observational studies, and a recent meta‐analysis has indicated that PAI‐1 4G/5G polymorphism may serve as a genetic biomarker for atherosclerotic diseases.^14^, ^15^, ^16^

To better understand the role of PAI‐1 in the outcome of stroke thrombolytic therapy, in the present study, we measured PAI‐1 activity and antigen levels during the course of thrombolysis in a cohort of consecutive acute ischemic stroke patients and studied the association of PAI‐1 levels and PAI‐1 4G/5G polymorphism with the outcome and safety of therapy.

## Materials and Methods

### Patients

Consecutive acute IS patients were enrolled in the study at the Department of Neurology, University of Debrecen, Hungary. Patient enrollment started in March 2011 and lasted till December 2012. Inclusion and exclusion criteria of patients included the standard criteria for rt‐PA administration of the 2008 ESO guideline.^17^ All patients underwent thrombolysis within the 4.5 h therapeutic time window using rt‐PA according to standard protocols.^17^ As at the time of patient enrollment mechanical thrombectomy was not yet available, none of the patients received such treatment. Baseline characteristics of the patient cohort have been published earlier.^18^ The presence of IS was diagnosed based on clinical symptoms, brain imaging using non‐contrast computerized tomography (NCCT) scan, and CT angiography (CTA). A control NCCT was performed for every patient 24 h after the event. Alberta Stroke Program Early CT Scores (ASPECTS) were calculated at both NCCT examinations by four independent radiologists.^19^ Stroke severity was determined by NIHSS (National Institutes of Health Stroke Scale) on admission, days 1 and 7 after therapy.^20^ Trial of ORG 10172 in Acute Stroke Treatment (TOAST) criteria was used to identify the etiology of stroke.^21^ Hemorrhagic transformation of stroke was classified as symptomatic or asymptomatic intracranial hemorrhage (ICH) using the European Cooperative Acute Stroke Study (ECASS) II criteria.^22^ Volume of post‐lysis intracerebral hemorrhage was calculated using 24h NCCT scans as described previously.^23^ Short‐term outcome was assessed at day 1 after the thrombolysis. A decrease in NIHSS score by at least 4 points or to 0 was defined as favourable outcome, while an increase in NIHSS score by at least 4 points was defined as poor outcome.^24^, ^25^ Long‐term outcome was defined according to the modified Rankin Scale (mRS) at 90 days. Patients with mRS 0–2 were defined as having favourable long‐term outcome.^26^

### Blood sampling and laboratory measurements

Peripheral blood samples were taken before the initiation of rt‐PA infusion, immediately after the administration of rt‐PA infusion (e.g., approximately 1 h after the initiation of therapy) and 24 h after thrombolysis. From the blood samples taken on admission, routine laboratory examinations were performed (ions, glucose levels, renal and liver function tests, high‐sensitivity C‐reactive protein (hsCRP), complete blood count) by standard methods (Roche Diagnostics, Mannheim, Germany and Sysmex Europe GmbH, Hamburg, Germany). Screening tests of coagulation (prothrombin time, activated partial thromboplastin time and thrombin time) were performed on a BCS coagulometer using routine methods (Siemens Healthcare Diagnostic Products, Marburg, Germany). For the examination of the PAI‐1, blood samples were drawn into Vacuette CTAD (sodium citrate, theophylline, adenosine, and dipyridamole anticoagulation) tubes. Blood samples were processed immediately and were centrifuged at 1220 g, room temperature for 15 min. Plasma aliquots were labelled with a code and stored at −70°C until further analysis. All measurements were performed by investigators blinded to patient identification and clinical data. PAI‐1 activity and antigen levels were measured using Technozym PAI‐1 Actibind ELISA and Technozym PAI‐1 Antigen ELISA assays, respectively, according to the manufacturer’s instructions. The Actibind assay exclusively measures free, active PAI‐1 (reference range: 1–7 U/mL as provided by the manufacturer). The Technozym PAI‐1 Antigen assay measures free, complexed, and latent PAI (normal range: 7–43 ng/mL as provided by the manufacturer). Other forms of PAI‐1 or other plasminogen activator inhibitors have no effect on the assays.

Genomic DNA was extracted from the buffy coat of blood samples using standard methods (QIAamp DNA Blood Mini Kit, Qiagen, Hilden, Germany). LightMixτ^®^ PAI‐1 4G/5G kit was used to detect the 4G/5G polymorphism in the promoter region of PAI‐1 using a LightCycler^®^ 480 instrument (Roche Diagmostics GmbH, Mannheim, Germany).

### Statistical analysis

Statistical analysis was performed using Stata 12 (Stata Corp, College Station, TX), the Statistical Package for Social Sciences (SPSS, Release 22.0, Chicago, IL), and GraphPad Prism 5.0 (GraphPad Prism Inc., La Jolla, CA). Shapiro–Wilk test was used to assess the normality of the data. Student’s t test or Mann–Whitney U test was performed for two‐group analyses. ANOVA with Bonferroni post hoc test or Kruskal–Wallis analysis with Dunn–Bonferroni post hoc test was applied for multiple comparisons. Pearson’s or Spearman’s correlation coefficient was used to determine the strength of correlation between PAI‐1 levels and other continuous variables. Differences between categorical variables were assessed by chi‐squaredtest or Fisher’s exact test. Binary backward logistic regression model was used to determine whether the presence of 5G/5G genotype is an independent predictor of ICH after thrombolysis. Variables were selected for entering the multivariate model based on the results of univariate analyses, correlation, and literature data. Results of the logistic regression analysis were expressed as odds ratio (OR) and 95% confidence interval (CI). A *P*‐value of <0.05 was considered statistically significant.

### Informed consent

The Ethics Committee of the University of Debrecen, Hungary approved the study. The study protocol conformed to the ethical guidelines of the 1975 Declaration of Helsinki. All patients or their relatives provided written informed consent.

## Results

### Baseline characteristics of enrolled patients according to their PAI‐1 4G/5G genotype

Baseline characteristics of enrolled patients are listed in Table 1. Among the 131 patients enrolled, 31 subjects had PAI‐1 5G/5G genotype. Baseline clinical or laboratory characteristics were not significantly different in PAI‐1 5G/5G homozygotes versus PAI‐1 4G carriers except for significantly more post‐lysis hemorrhagic events in PAI‐1 5G/5G homozygotes (frequency of hemorrhage: 19.35% vs. 7% in PAI 5G/5G homozygotes vs. PAI‐1 4G carriers, *P* = 0.036).

**Table 1:** Baseline characteristics of enrolled patients according to their PAI‐1 4G/5G genotype.

	5G/5G	4G/5G and 4G/4G	*P*
Number of patients	31	100	
Age (years), mean (SD)	69.9 (13.6)	68.8 (11.8)	0.547
Male, *n* (%)	17 (54.8)	62 (62.0)	0.476
Cerebrovascular risk factors, *n* (%)			
Arterial hypertension	26 (83.9)	74 (74.0)	0.259
Atrial fibrillation	7 (22.6)	28 (28.0)	0.551
Previous stroke	8 (26.7)	34 (34.3)	0.432
Hyperlipidemia	19 (61.3)	62 (62.0)	0.943
Diabetes mellitus	9 (29.0)	30 (30.0)	0.918
BMI	28.24 (±4.80)	27.65 (±5.07)	0.562
Smoking, *n* (%)			
Non‐smoker	17 (54.8)	52 (52.0)	
Previous smoker	3 (9.7)	13 (13.0)	0.951
Current smoker	7 (22.6)	24 (24.0)	
Undetermined	4 (12.9)	11(11.0)	
Duration of thrombolysis, median (IQR)	60.0 (60.0–63.5)	60.0 (60.0‐63.5)	0.444
Time‐to‐treatment (min), median (IQR)	158 (133–203)	153 (125–177)	0.231
rt‐PA dose (mg), mean (SD)	69.5 (15.3)	67.7 (14.9)	0.732
Medication at enrollment, *n* (%)			
Antihypertensive therapy	22 (75.9)	71 (71.7)	0.660
Antiplatelet drug	14 (45.2)	44 (44.9)	0.980
Anticoagulant drug	3 (10.0)	4 (4.04)	0.207
Lipid lowering therapy	9 (31.0)	29 (29.3)	0.857
Antidiabetic therapy	3 (10.0)	13 (13.3)	0.636
Laboratory measurements, median (IQR)			
INR	0.98 (0.94–1.05)	0.99 (0.95–1.03)	0.905
APTT (sec)	28.6 (26.6–32.1)	28.35 (26.1–32.15)	0.920
WBC (G/L)	6.59 (5.76–8.66)	7.63 (6.24–9.06)	0.330
Platelets (G/L)	188 (169–253)	212 (171–255)	0.529
Serum glucose (mmol/L)	6.4 (5.7–8.7)	6.5 (5.5–7.4)	0.560
hsCRP (mg/L)	3.31 (1.50–6.63)	3.01 (1.70–5.42)	0.701
Creatinine (*μ*mol/L)	67 (60–100)	81 (67–97)	0.180
Admission NIHSS, median (IQR)	9 (5–13)	8 (5–14)	0.693
Stroke etiology (TOAST), *n* (%)			
Large‐artery atherosclerosis	16 (51.6)	33 (33.0)	
Small‐vessel occlusion	3 (9.7)	10 (10.0)	0.292
Cardioembolic	5 (16.1)	22 (22.0)	
Other/undetermined	7 (22.6)	35 (35.0)	
Imaging data, *n* (%)			
ASPECTS on admission			
0–7	1 (3.8)	3 (4.5)	
8–10	25 (96.2)	64 (95.5)	0.893
ASPECTS at 24 h after thrombolysis			
0–7	10 (38.5)	28 (41.8)	
8–10	16 (61.5)	39 (58.2)	0.769
Outcomes, *n* (%)			
Short‐term outcome (24 h)			
Good outcome (−4 points or 0)	10 (32.2)	28 (28.0)	
Unchanged status (±3 points)	9 (29.0)	49 (49.0)	0.496
Poor outcome (+4 points)	4 (12.9)	12 (12.0)	
Undetermined	2 (6.5)	4 (4.0)	
Long‐term outcome (90 days)			
mRS 0–2	9 (29.0)	48 (48.0)	
mRS 3–6	15 (48.4)	36 (36.0)	0.176
Undetermined	7 (22.6)	16 (16.0)	
Intracranial hemorrhage (ECASS II)			
No hemorrhage	25 (80.65)	93 (93)	
aSICH	2 (6.45)	5 (5.0)	0.036
SICH	4 (12.9)	2 (2.0)	

APTT, activated partial thromboplastin time; aSICH, asymptomatic intracerebral hemorrhage; ASPECTS, The Alberta Stroke Program early CT score; ECASS II, European Co‐operative Acute Stroke Study‐II; hsCRP, high‐sensitive CRP; INR, international normalized ratio; IQR, interquartile range; mRS, modified Rankin Scale; NIHSS, National Institutes of Health Stroke Scale; rt‐PA, recombinant tissue plasminogen activator; SD, standard deviation; SICH, symptomatic intracerebral hemorrhage; TOAST, Trial of ORG 10172 in Acute Stroke Treatment; WBC, white blood cell.

### PAI‐1 levels during thrombolysis

**Figure 1:**
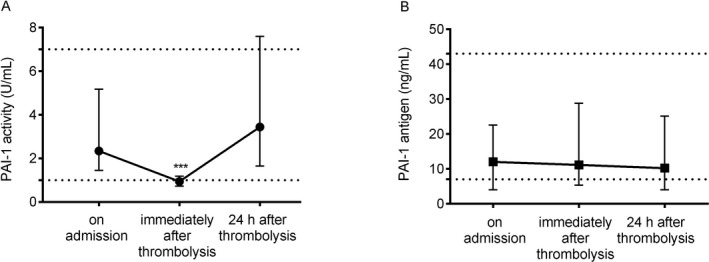
Plasminogen activator inhibitor‐1 (PAI‐1) activity (A) and antigen (B) levels as assessed on admission, immediately after thrombolysis, and 24 h after thrombolysis in acute ischemic stroke patients. Solid symbols represent median values, whiskers indicate interquartile ranges. Upper and lower limits of reference intervals are indicated by dotted lines. ***P < 0.0001 (Kruskal–Wallis analysis with Dunn–Bonferroni post hoc test).

PAI‐1 activity levels dropped transiently immediately after thrombolysis (Fig. 1A). As compared to admission PAI‐1 activity levels, a highly significant reduction was observed; the median value of PAI‐1 activity was below the lower limit of the reference interval when measured immediately after thrombolysis (PAI‐1 activity on admission: median: 2.34, IQR: 1.46–5.17 U/mL; immediately after thrombolysis: median: 0.94, IQR: 0.73–1.18 U/mL). The narrow interquartile range of PAI‐1 activity level immediately after thrombolysis is to be noted. Twenty‐four hours after thrombolysis PAI‐1, activity showed a substantial elevation (median: 3.44, IQR: 1.65–7.60 U/L). As opposed to PAI‐1 activity, PAI‐1 antigen levels remained unchanged during the course of thrombolysis (Fig 1B). Best correlation between PAI‐1 activity and antigen levels was observed 24 h after thrombolysis (Spearman *r*: 0.539, *P* < 0.001; *r*: 0.355, *P* < 0.001, and *r*: 0.752, *P* < 0.001, on admission, immediately after thrombolysis, and 24 h after thrombolysis, respectively).

PAI‐1 4G/5G polymorphism had no effect on PAI‐1 activity and PAI‐1 antigen levels as measured during the course of thrombolysis in this cohort (Table S1). Among the baseline clinical and laboratory parameters (e.g., age, BMI, CRP, etc. as listed in Table 1), PAI‐1 levels showed a fair correlation with BMI and CRP, particularly in samples measured 24 h after thrombolysis (PAI‐1 activity 24 h after thrombolysis and BMI: Spearman *r*: 0.338, *P* < 0.001; PAI‐1 activity 24 h after thrombolysis and CRP: Spearman r: 0.418, *P* < 0.001). PAI‐1 levels showed insignificant diurnal variation in this patient cohort (median admission PAI‐1 activity from 0:00 to 12:00 am: 2.81, IQR: 1.48–5.97 U/mL vs. PAI‐1 activity between 12:01–23:59: 2.09, IQR: 1.45–4.68 U/mL, *P* = 0.363).

### Association of PAI‐1 activity and antigen levels with stroke severity, etiology, and outcomes

PAI‐1 activity and antigen levels on admission showed no association with stroke severity or etiology (Table S2). Admission and 24 h post‐lysis, PAI‐1 activity levels were significantly higher in patients presenting with worse ASPECTS (<7) at 24 h after thrombolysis (Table 2). PAI‐1 antigen levels were also significantly higher on admission and at 1 h post‐lysis in those with worse ASPECTS at 24 h (Table 2). Despite these associations, PAI‐1 activity and antigen levels as measured during the course of thrombolysis showed no association with post‐lysis hemorrhage, short‐term or long‐term functional outcomes (Table 3 and Table 4). PAI‐1 activity and antigen levels at any given time point showed no correlation with post‐lysis intracerebral hematoma volume (data not shown).

**Table 2:** PAI‐1 activity and antigen levels according to ASPECTS on admission and at 24 h after thrombolysis.

	ASPECTS on admission			ASPECTS 24 h after thrombolysis		
	10‐8 (*n* = 89)	7‐0 (*n* = 4)	*P*	10‐8 (*n* = 55)	7‐0 (*n* = 38)	*P*
PAI‐1 activity (U/mL), median (IQR)						
On admission	2.44 (1.45–4.72)	4.78 (2.30–14.46)	0.262	1.91 (1.38–3.79)	3.43 (1.79–6.76)	0.038
1 h after thrombolysis	0.92 (0.72–1.14)	1.61 (0.59–3.70)	0.693	0.90 (0.69–1.13)	0.98 (0.72–1.25)	0.233
24 h after thrombolysis	3.80 (1.82–7.85)	3.42 (2.84–7.25)	0.856	2.94 (1.80–6.00)	5.17 (2.19–10.97)	0.084
PAI‐1 antigen (ng/mL), median IQR						
On admission	12.05 (4.03–23.92)	15.50 (7.09–39.66)	0.640	7.33 (3.99–20.24)	14.08 (7.61–26.31)	0.041
1 h after thrombolysis	11.12 (5.52–28.16)	12.22 (4.6–31.43)	0.802	9.53 (3.99–18.91)	19.23 (6.15–43.63)	0.023
24 h after thrombolysis	12.09 (3.99–26.26)	4.46 (3.99–8.51)	0.169	8.48 (3.99–19.96)	12.77 (3.99–34.11)	0.291

ASPECTS, The Alberta Stroke Program early CT score; IQR, interquartile range; PAI‐1, plasminogen activator inhibitor −1.

**Table 3:** PAI‐1 activity levels according to thrombolysis safety and outcomes.

PAI‐1 activity (U/mL), median (IQR)						
	On admission	*P*	1 h after thrombolysis	*P*	24 h after thrombolysis	*P*
Intracranial hemorrhage (ECASS II)						
No hemorrhage	2.47 (1.48–5.17)		0.92 (0.73–1.16)		3.44 (1.71–7.66)	
aSICH	1.73 (1.26–14.23)	0.667	1.04 (0.94–1.25)	0.210	1.57 (0.9–7.54)	
SICH	2.19 (0.88–3.24)		1.02 (0.97–1.62)		6.26 (3.24–13.08)	0.345
Short‐term outcome (24 h)						
Good outcome (− 4 points or 0)	2.74 (1.59–6.82)		1.04 (0.77–1.15)		3.80 (2.23–7.27)	
Unchanged status (±3 points)	2.44 (1.45–4.47)		0.88 (0.65–1.2)		2.96 (1.58–7.54)	
Poor outcome (+4 points)	2.04 (1.47–5.15)	0.730	0.97 (0.87–1.08)	0.364	5.21 (1.52–12.31)	0.326
Undetermined	1.31 (1.44–4.72)		0.96 (0.8–1.24)		1.52 (1.16–1.82)	
Functional outcome at 90 days						
mRS 0–2	2.47 (1.64–3.94)		0.90 (0.73–1.14)		2.86 (1.54–5.78)	
mRS 3–6	2.13 (1.31–5.97)	0.845	0.98 (0.80–1.21)	0.478	4.84 (1.66–11.44)	0.091
Undetermined	2.44 (1.26–5.19)		1.08 (0.65–1.36)		4.20 (2.97–7.29)	

aSICH, asymptomatic intracerebral hemorrhage; ECASS II, European Co‐operative Acute Stroke Study‐II; IQR, interquartile range, mRS, modified Rankin Scale; PAI‐1, plasminogen activator inhibitor −1; SICH, symptomatic intracerebral hemorrhage

**Table 4:** PAI‐1 antigen levels according to thrombolysis safety and outcomes.

PAI‐1 antigen (ng/mL), median (IQR)						
	On admission	*P*	1 h after thrombolysis	*P*	24 h after thrombolysis	*P*
Intracranial hemorrhage (ECASS II)						
No hemorrhage	12.00 (3.99–23.23)		11.73 (5.31–29.99)		9.51 (3.99–21.36)	
aSICH	10.54 (4.03–17.04)	0.763	9.25 (3.99–22.05)	0.935	5.51 (3.99–23.83)	0.615
SICH	12.00 (3.99–23.23)		10.22 (6.60–18.45)		5.76 (0–52.57)	
Short‐term outcome (24 h)						
Good outcome (−4 points or 0)	18.13 (4.95–28.67)		12.31 (5.16–22.65)		8.34 (3.99–20.10)	
Unchanged status (±3 points)	10.46 (3.99–17.71)		9.87 (3.99–34.78)		9.51 (3.99–28.92)	
Poor outcome (+4 points)	12.00 (3.99–24.39)	0.471	9.74 (6.15–18.94)	0.816	11.59 (3.99–19.96)	0.713
Undetermined	9.42 (5.88–15.11)		18.24 (10.75–37.42)		4.01 (3.99–8.06)	
Functional outcome at 90 days						
mRS 0–2	7.24 (3.99–19.96)		9.93 (3.99–21.46)		7.38 (3.99–19.75)	
mRS 3–6	12.73 (4.03–23.92)	0.441	14.69 (5.87–35.47)	0.398	12.58 (3.99–36.03)	0.431
Undetermined	13.09 (5.68–25.48)		12.61 (5.66–37.69)		10.56 (3.99–12.56)	

aSICH, asymptomatic intracerebral hemorrhage; ECASS II, European Co‐operative Acute Stroke Study‐II; IQR, interquartile range, mRS, modified Rankin Scale; PAI‐1, plasminogen activator inhibitor −1; SICH, symptomatic intracerebral hemorrhage.

### PAI‐1 4G/5G genotype is an independent predictor of ICH

**Figure 2:**
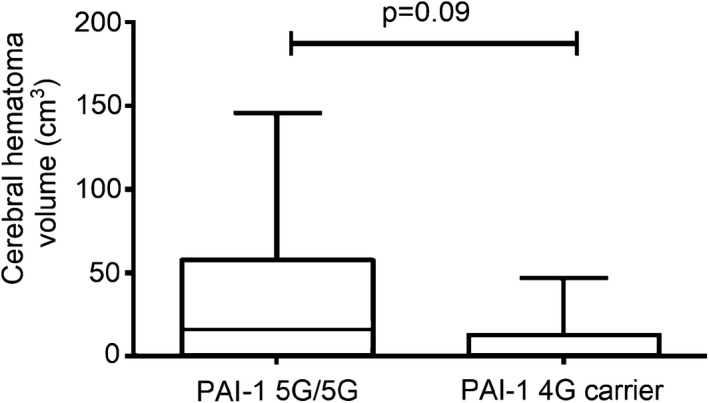
Volume of post‐lysis cerebral hemorrhage according to PAI‐1 4G/5G polymorphism in acute ischemic stroke patients. Box‐whiskers plots indicate median, interquartile range, and total range. Six patients are included in both groups. Volume of hematoma was not calculated in case of one patient who suffered subarachnoideal hemorrhage.

Patients who suffered post‐lysis ICH (*n* = 13) in this cohort had significantly lower BMI (Table 5). The frequency of hypertension and hyperlipidemia was significantly lower in this group as compared to those without post‐lysis bleeding complications. In a binary backward logistic regression model including age, gender, BMI, NIHSS on admission, hypertension, hyperlipidemia and PAI‐1 4G/5G genotype, PAI‐1 5G/5G genotype was revealed as a significant, independent risk factor for post‐lysis ICH (Table 6). The risk conferred by PAI‐1 5G/5G for post‐lysis ICH was almost fivefold (OR: 4.75, 95%CI: 1.18–19.06, *P* = 0.028). Patients with PAI‐1 5G/5G genotype showed a trend toward larger post‐lysis intracerebral hemorrhage volume as compared to PAI‐1 4G carriers (median: 16.82, IQR: 1.46–58.16 cm^3^ vs. median: 0.67, IQR: 0.26–13.55 cm^3^, *P* = 0.09; Fig. 2).

**Table 5:** Characteristics of enrolled patients according to the presence of hemorrhagic transformation after thrombolysis.

	No intracranial hemorrhage	Intracranial hemorrhage	*P*
Number of patients	118	13	
Age (years), mean (SD)	70 (69.5)	64 (64.7)	0.548
Male *n*%	71 (60.2)	8 (61.5)	0.924
Cerebrovascular risk factors			
Arterial hypertension	94 (79.7)	6 (46.2)	0.007
Atrial fibrillation	31 (26.3)	4 (30.8)	0.746
Previous stroke	42 (36.2)	0 (0)	–
Hyperlipidemia	78 (66.1)	3 (23.1)	0.005
Diabetes mellitus	38 (32.2)	1 (7.7)	0.107
BMI	28.12 (±5.02)	24.97 (±3.82)	0.031
Smoking *n*%			
Non‐smoker	63 (53.4)	6 (46.2)	
Previous smoker	14 (11.9)	2 (15.4)	0.358
Current smoker	26 (22.0)	5 (38.5)	
Undetermined	15 (12.7)	0 (0)	
Duration of thrombolysis, median (IQR)	60 (60–65)	60 (60–61.5)	0.444
Time‐to‐treatment (min), median (IQR)	155 (125–180)	144 (132–177)	0.231
rt‐PA dose (mg), mean (SD)	69 (68.6)	65 (63.5)	0.732
Baseline NIHSS	8 (5–14)	12 (8–16)	0.189
Medication at enrollment, *n* (%)			
Antihypertensive therapy	87 (75.7)	6 (46.2)	0.024
Antiplatelet drug	54 (46.6)	4 (30.8)	0.381
Anticoagulant drug	6 (5.2)	1 (7.7)	0.533
Lipid lowering therapy	37 (32.2)	1 (7.7)	0.106
Antidiabetic therapy	13 (11)	0 (0)	0.151
Stroke etiology (TOAST), *n*%			
Large‐artery atherosclerosis	43 (36.4)	6 (46.2)	
Small‐vessel occlusion	11 (9.3)	2 (15.4)	0.155
Cardioembolic	23 (19.5)	4 (30.8)	
Other/undetermined	41 (34.8)	1 (7.7)	
Basic laboratory measurements, median (IQR)			
INR	0.98 (0.94–1.02)	1.07 (0.95–1.09)	0.905
APTT (sec)	28.4 (26–32.1)	28.6 (28.1–32.4)	0.920
WBC (G/L)	7.42 (6.08–8.61)	8.08 (6.49–9.41)	0.331
Platelets (G/L)	208 (171–254)	194 (169–260)	0.530
Serum glucose (mmol/l)	6.45 (5.5–7.95)	6.5 (5.9–7.4)	0.560
hsCRP (mg/L)	3.02 (1.7–5.8)	3.25 (1.6–6.6)	0.701
Creatinine (umol/L)	82.5 (65–98)	66 (57–71)	0.181
PAI‐1 measurements			
PAI‐1 activity (U/mL), median (IQR)			
on admission	2.47 (1.48–5.17)	1.91 (1.26–3.24)	0.507
immediately after thrombolysis	0.92 (0.73–1.16)	1.03 (0.97–1.25)	0.843
24 h after thrombolysis	9.51 (3.99–21.36)	5.51 (3.99–23.83)	0.274
PAI‐1 antigen (ng/mL), median (IQR)			
on admission	12 (3.99–23.23)	11.48 (6.44–18.52)	0.370
immediately after thrombolysis	11.73 (5.31–29.99)	9.74 (5.35–19.66)	0.752
24 h after thrombolysis	9.51 (3.99–21.36)	5.51 (3.99–23.83)	0.274
PAI‐1 5G homozygous, *n* (%)	25 (21.19)	6 (46.15)	0.044

APTT, activated partial thromboplastin time; hsCRP, high‐sensitive CRP; INR, international normalized ratio; IQR, interquartile range, PAI‐1, plasminogen activator inhibitor −1; rt‐PA, recombinant tissue plasminogen activator; SD, standard deviation; TOAST, Trial of ORG 10172 in Acute Stroke Treatment; WBC white blood cell.

**Table 6:** Independent predictors of post‐lysis intracranial hemorrhage in the studied cohort.

	OR	95% CI	*P*
BMI	0.89	0.77–1.04	0.149
Arterial hypertension	0.28	0.71–1.12	0.073
Hyperlipidemia	0.21	0.05–0.88	0.033
PAI‐1 5G/5G genotype	4.75	1.18–19.06	0.028

Backward multiple regression model included age, sex, NIHSS on admission, BMI, history of arterial hypertension, history of hyperlipidemia, PAI‐1 5G/5G genotype. OR, odds ratio; CI, confidence interval; BMI, body mass index; PAI‐1, plasminogen activator inhibitor −1.

## Discussion

Despite the fact that PAI‐1 is the most effective natural inhibitor of t‐PA, to date, its role in the outcome of ischemic stroke thrombolytic therapy has not been fully elucidated.^27^ A major finding of this study is that PAI‐1 5G/5G genotype confers an independent, significant risk for post‐lysis ICH. In a most recent systematic review on potential prognostic coagulation biomarkers in acute ischemic stroke, PAI‐1 was mentioned as one of the most promising candidates when assessed before the start of reperfusion treatment.^27^ Interestingly, to date, no study has been published where levels of PAI‐1 during thrombolysis and PAI‐1 4G/5G polymorphism were simultaneously investigated exploring potential associations with the safety and outcome of stroke thrombolysis in humans. Here we show that in this studied patient cohort, 46.15% of patients suffering post‐lysis ICH were PAI‐1 5G homozygous individuals. In a multiple logistic regression model including all potential conventional risk factors for post‐lysis hemorrhage, the presence of PAI‐1 5G/5G genotype conferred the strongest independent risk for hemorrhagic transformation (OR: 4.75 95%CI: 1.18–19.06, *P* = 0.028). Moreover, a trend toward larger hematomas in patients with PAI‐1 5G/5G genotype was observed, which further supports the potential role of this polymorphism in the pathophysiology of post‐lysis hemorrhage.

The contributing effect of PAI‐1 5G/5G genotype to post‐lysis hemorrhage might be in theory related to its effect on plasma PAI‐1 levels regulating intravascular fibrinolysis or to a local effect of PAI‐1 levels in the brain parenchyma limiting excessive t‐PA activity. To date, published studies on plasma PAI‐1 levels before or during thrombolysis have included relatively few patients, and in these studies either PAI‐1 activity or antigen levels were determined; thus results are difficult to compare, and are often inconclusive or contradictory.^28^, ^29^, ^30^, ^31^ Interestingly, in our study PAI‐1 4G/5G polymorphism did not seem to have a major influence on plasma PAI‐1 levels, which is in line with few previous reports.^32^, ^33^, ^34^, ^35^ The contributing effect of PAI‐1 5G/5G genotype to post‐lysis ICH via lower levels of PAI‐1 at the site of the intracerebral lesion is nevertheless biologically plausible. Local PAI‐1 levels in the thrombus might be essentially different from peripheral levels. Moreover, following acute stroke intravascular PAI‐1 levels show a rising tendency in animal models,^36^ and the same phenomenon was confirmed by this study in humans. Known influencing factors of PAI‐1 levels (e.g., BMI and inflammation) were confirmed by the present study. Interestingly, patients who suffered post‐lysis ICH were found to have significantly lower BMI. Although a potential association between low PAI‐1 levels, low BMI, and post‐lysis hemorrhage is intriguing, according to the backward regression model used in this study, BMI was not conferred as an independent risk for post‐lysis hemorrhage and thus this line of investigations should be carried out in larger patient cohorts.

The association of PAI‐1 5G/5G genotype with post‐lysis intracerebral bleeding might extend beyond intravascular fibrinolysis. PAI‐1, derived from astrocytes, can reduce toxicity and neuronal cell damage by limiting excessive t‐PA activity in the brain parenchyma.^37^, ^38^, ^39^ Besides astrocytes, brain endothelial cells and pericytes also express PAI‐1.^40^ PAI‐1 released from brain endothelial cells and pericytes is known to prevent blood–brain barrier disruption.^41^ As PAI‐1 4G/5G polymorphism affects PAI‐1 transcriptional activity in human astrocytes,^42^ the potential contributing effect of PAI‐1 5G/5G genotype to post‐lysis hemorrhage might be related to a direct, local exacerbation of brain damage rather than a regulating effect on plasma PAI‐1 levels. Given the complex role of PAI‐1 in stroke pathophysiology, both theories are plausible and warrant further experimental studies.

Here we also show that PAI‐1 activity significantly decreases upon thrombolytic treatment, while at the same time no change is observed in PAI‐1 antigen levels (including free, t‐PA‐complexed, and latent forms). In this cohort, median PAI‐1 activity immediately after thrombolysis decreased below the lower limit of reference, which indicates that PAI‐1 efficiently inhibits excess rt‐PA during thrombolytic therapy. Nevertheless, absolute values of PAI‐1 activity and PAI‐1 antigen did not show a significant association with the inefficacy or the safety of the treatment. It must be noted, however, that significantly higher on admission and 1h post‐lysis PAI‐1 antigen levels were detected in patients in whom imaging data suggested a more severe lesion at 24 h post‐event (ASPECTS < 7). In this group of patients with 24 h ASPECTS of less than 7, median PAI‐1 antigen levels on admission and at 1h post‐lysis were twice as high as compared to those with better scores. A similar, significant difference in PAI‐1 activity levels on admission was observed between the two groups of different stroke severity as judged by imaging scores. These results indicate that an elevated PAI‐1 activity/antigen result on admission might predict the possibility of a more severe definitive lesion as detected by CT scans at 24 h post‐event. Such association of PAI‐levels with imaging results has not been shown before. Interestingly, the median value of PAI‐1 activity at 24 h post‐lysis showed a clear trend for increase in patients with worse long‐term functional outcomes (mRS 3–6); however, the association did not reach statistical significance in this patient cohort (*P* = 0.091).

### Limitations

Similar to most observational clinical studies, our study has limitations as well. The sample size is limited; however, as compared to other published studies measuring fibrinolysis inhibitors in stroke patients on admission or particularly at multiple time points during the course of thrombolysis, it is the largest study as yet.^28^, ^29^, ^30^, ^31^ Due to the limited number of patients with post‐lysis ICH, despite the significant associations found, results presented here must be confirmed and validated by larger studies. Although the difference was nonsignificant, it must be noted that large‐artery atherosclerosis was more frequent among PAI‐1 5G/5G patients, that could have reached statistical significance with a larger sample size. Moreover, large‐artery atherosclerosis and cardioembolic type of strokes were more frequent in patients experiencing post‐lysis bleeding. Being single‐centered, our study had the advantages of uniform sample handling and uniform patient care, but, as the center recruits patients from a relatively large geographic area, unfortunately a proportion of patients (23/131, 17.5%) were lost to long‐term follow‐up. This percentage of follow‐up drop‐out is comparable to that observed in other studies involving post‐stroke patients;^27^ however, it might have influenced the results regarding long‐term outcomes to a certain extent and thus larger clinical studies are needed to confirm our data.

## Conclusions

Here we demonstrate that PAI‐1 5G/5G genotype is associated with a significant, independent risk for developing ICH in post‐lysis stroke patients. Further studies are warranted to validate this finding and to find out whether patients with PAI‐1 5G/5G genotype might benefit from alternative therapeutic strategies.

## Conflict of Interest

The authors declare that there are no competing interests concerning this work.

## Author Contributions

L.C. and Z.B. designed the study. I.S., R.K.C‐K., L.I.L., and E.B. were involved in sample collection and source data preparation. E.G.S. and F.S. performed the measurements. I.S., A.N., and Z.B. analyzed the data, designed and performed the statistical analysis. I.S. and Z.B. wrote the paper. All authors agreed to the final version of the manuscript.

## Supporting information


**Table S1.** PAI‐1 activity and antigen levels according to PAI‐1 4G/5G polymorphism.

**Table S2.** PAI‐1 activity and antigen levels according to stroke severity on admission and stroke etiology.
