# Supplementary material for: PAI‐1 5G/5G genotype is an independent risk of intracranial hemorrhage in post‐lysis stroke patients
Source: Ann Clin Transl Neurol. 2019 Oct 21;6(11):2240–50. doi: 10.1002/acn3.50923 (PMC6856768; doi:10.1002/acn3.50923)
Supplement: Supplementary file 1 — Table S1. PAI‐1 activity and antigen levels according to PAI‐1 4G/5G polymorphism. [file ACN3-6-2240-s001.docx]

|  | 5G/5G | 4G/5G and 4G/4G | P value |
| --- | --- | --- | --- |
| PAI-1 activity (U/mL), median (IQR)  on admission  immediately after thrombolysis  24h after thrombolysis  PAI-1 antigen (ng/mL), median (IQR)  on admission  immediately after thrombolysis  24h after thrombolysis | 2.34 (0.91-5.97)  0.94 (0.87-1.14)  3.56 (1.91-7.29)  12.05 (5.01-25.43)  9.04 (3.99-18.3)  10.51 (5.51-19.96) | 2.34 (1.50-5.15)  0.93 (0.73-1.21)  3.46 (1.59-8.09)  11.63 (3.99-22.46)  11.73 (5.59-29.99)  8.34 (3.99-24.27) | 0.398  0.825  0.839  0.558  0.405  0.280 |
|  |  |  |  |

Supplementary Table 1. PAI-1 activity and antigen levels according to PAI-1 4G/5G polymorphism

IQR, interquartile range; PAI-1, plasminogen activator inhibitor-1
