# Supplementary material for: PAI‐1 5G/5G genotype is an independent risk of intracranial hemorrhage in post‐lysis stroke patients
Source: Ann Clin Transl Neurol. 2019 Oct 21;6(11):2240–50. doi: 10.1002/acn3.50923 (PMC6856768; doi:10.1002/acn3.50923)
Supplement: Supplementary file 2 — Table S2. PAI‐1 activity and antigen levels according to stroke severity on admission and stroke etiology. [file ACN3-6-2240-s002.docx]

|  | PAI-1 activity on admission (U/ml),  median (IQR) | P | PAI-1 antigen on admission (ng/ml),  median (IQR) | P |
| --- | --- | --- | --- | --- |
| Stroke severity on admission  NIHSS 0-5 (n=36)  NIHSS 6-10 (n=46)  NIHSS 11-16 (n=29)  NIHSS >16 (n=17)  Undetermined (n=3)  Stroke etiology (TOAST)  Large-artery atherosclerosis (n=49)  Small-vessel occlusion (n=13)  Cardioembolic (n=27)  Other/undetermined (n=42) | 2.34 (1.57-6.54)  2.61 (1.71-5.15)  2.98 (1.62-6.44)  1.36 (0.87-2.44)  1.38 (1.04-4.72)  2.74 (1.47-4.57)  2.50 (1.82-5.28)  1.91 (1.28-6.05)  2.24 (1.50-6.82) | 0.107  0.963 | 6.65 (3.99-17.96)  15.70 (6.27-23.92)  13.36 (4.50-32.18)  13.40 (5.46-19.75)  9.01 (5.88-9.82)  11.07 (5.01-17.71)  6.30 (3.99-21.40)  9.01 (3.99-24.39)  16.16 (5.44-23.92) | 0.282  0.663 |

IQR**,** interquartile range; NIHSS, National Institutes of Health Stroke Scale; PAI-1, plasminogen activator inhibitor-1; TOAST, Trial of ORG 10172 in Acute Stroke Treatment

Supplementary Table 2. PAI-1 activity and antigen levels according to stroke severity on admission and stroke etiology

Stroke severity of patients was classified according to previous literature ^1,2^.

References

1. Logallo N, Kvistad CE, Naess H, Waje-Andreassen U, Thomassen L. Mild stroke: safety and outcome in patients receiving thrombolysis. *Acta Neurol Scand Suppl.* 2014(198):37-40.

2. Jovin TG, Chamorro A, Cobo E, et al. Thrombectomy within 8 hours after symptom onset in ischemic stroke. *N Engl J Med.* 2015;372(24):2296-2306.
